# Recruiting the right hemisphere: Sex differences in inter-hemispheric communication during semantic verbal fluency

**DOI:** 10.1016/j.bandl.2020.104814

**Published:** 2020-06-02

**Authors:** Andrea Scheuringer, Ti-Anni Harris, Belinda Pletzer

**Affiliations:** Department of Psychology, University of Salzburg, Salzburg, Austria, Centre for Cognitive Neuroscience, University of Salzburg, Salzburg, Austria

**Keywords:** Semantic verbal fluency, Verbal fluency strategies, Clustering, Switching, Sex differences, fMRI, Connectivity

## Abstract

Sex differences in cognitive functions are heavily debated. Recent work suggests that sex differences do stem from different processing strategies utilized by men and women. While these processing strategies are likely reflected in different brain networks, so far the link between brain networks and processing strategies remains speculative. In the present study we seek for the first time to link sex differences in brain activation patterns to sex differences in processing strategies utilizing a semantic verbal fluency task in a large sample of 35 men and 35 women, all scanned thrice. For verbal fluency, strategies of clustering and switching have been described. Our results show that men show higher activation in the brain network supporting clustering, while women show higher activation in the brain network supporting switching. Furthermore, converging evidence from activation results, lateralization indices and connectivity analyses suggests that men recruit the right hemisphere more strongly during clustering, but women during switching. These results may explain findings of differential performance and strategy-use in previous behavioral studies.

## 1 Introduction

Verbal fluency tasks require participants to generate words that correspond to a given criterion (i.e. belonging to a given semantic category or starting with a specific letter) within a given time-constraint (Lezak, 1995; Troyer, Moscovitch, & Winocur, 1997). Tests of verbal fluency have traditionally been employed in clinical settings to measure executive (dys-)functioning in patients with neurological damage or patients with neurodegenerative disorders (Lezak, 1995; Troyer et al., 1997). Longstanding research on verbal fluency suggest that these tests not only demand executive functioning but require numerous different cognitive processes, including working memory, self-monitoring, cognitive flexibility, lexical selection, phonetic encoding, word knowledge, verbal long-term memory and verbal intelligence (e.g. Ardila, Galeano, & Rosselli, 1998; Birn et al., 2010; Bolla, Lindgren, Bonaccorsy, & Bleecker, 1990; Cauthen, 1978; Daneman, 1991; Fisk & Sharp, 2004; Miller, 1984; Ruff, Light, Parker, & Levin, 1997; Salthouse, 1993; Shao, Janse, Visser, & Meyer, 2014; Unsworth, Spillers, & Brewer, 2011).

Given the idea that verbal fluency tests integrate various different cognitive functions, it is not surprising that verbal fluency tasks are accompanied by the activation of an extensive network of cortical and subcortical brain areas. While the (left) inferior frontal gyrus (IFG) was found to contribute strongest to verbal fluency, additional regions were observed to be recruited during verbal fluency: the dorsolateral prefrontal cortex (DLPFC), medial and lateral temporal areas, anterior cingulate gyrus (ACC), supplementary motor area (SMA), premotor cortex, the insula, and the cerebellum (e.g. Cuenod et al., 1995; Friedman et al., 1998; Frith, Friston, Liddle, & Frackowiak, 1991; Fu et al., 2002; Gauthier, Duyme, Zanca, & Capron, 2009; Gourovitch et al., 2000; Halari et al., 2006; Hugdahl et al., 1999; Lurito, Kareken, Lowe, Chen, & Mathews, 2000; Paulesu et al., 1997; Phelps, Hyder, Blamire, & Shulman, 1997; Pihlajamaki et al., 2000; Rueckert et al., 1994; Schlösser et al., 1998; for a meta-analysis: see Wagner, Sebastian, Lieb, Tüscher, & Tadić, 2014). Involvement of these areas is however dependent on the type of task used (i.e. phonemic vs. semantic fluency) (e.g.: Baldo, Schwartz, Wilkins, & Dronkers, 2006; Birn et al., 2010; Gourovitch et al., 2000; Mummery, Patterson, Hodges, & Wise, 1996; Pihlajamaki et al., 2000; for a meta-analysis see Wagner et al., 2014; for a review see Costafreda et al., 2006).

In phonemic fluency tasks participants are asked to generate words starting with a given letter (e.g. words starting with the letter ‘F’), whereas in semantic fluency tasks participants need to generate words corresponding to a given semantic category (e.g. ‘animals’) (Lezak, 1995; Troyer et al., 1997). While the two fluency tasks share some commonalities, they also require different cognitive processes and thus, partially rely on different brain regions (e.g. Baldo et al., 2006; Birn et al., 2010; Gourovitch et al., 2000; Mummery et al., 1996; Perret, 1974; Pihlajamaki et al., 2000; for a meta-analysis see Wagner et al., 2014; for a review see Costafreda et al., 2006). Whereas phonemic fluency is suggested to require mainly executive functioning and phonetic encoding, semantic fluency sets demands on semantic and lexical memory (e.g. Azuma, 2004; Baldo et al., 2006, Birn et al., 2010; Martin, Wiggs, Lalonde, & Mack, 1994; Perret, 1974; Rende, Ramsberger, & Miyake, 2002; Troyer et al., 1997; Unsworth et al., 2011). Several neuroimaging and lesion studies suggest that phonemic fluency primarily recruits frontal brain areas, while semantic fluency depends more strongly on temporal areas (Baldo et al., 2006; Baldo, Schwartz, Wilkins, & Dronkers, 2010; Benton, 1968; Birn et al., 2010; Butters, Granholm, Salmon, Grant, & Wolfe, 1987; Coslett, 1991; Crawford, Obonsawin, & Bremner, 1993; Gourovitch et al., 2000; Janowsky, Shimamura, Kritchevsky, & Squire, 1989; Martin et al., 1994; Milner & Petrides, 1984; Milner, 1964; Monsch, Bondi, Butters, & Paulsen, 1994; Moscovitch, 1994; Mummery et al., 1996; Newcombe & Russell, 1969; Patterson, Mack, Geldmacher, & Whitehouse, 1996; Perret, 1974).

Troyer et al. (1997) suggested that verbal fluency requires the cooperation of two different strategies, clustering and switching. The clustering strategy refers to the generation of words within one subcategory, described as a relative automatic process. The switching strategy reflects the generation of successive words not belonging to the same subcategory, requiring increased cognitive flexibility and reflecting a more effortful process (Troyer et al., 1997). It is proposed that participants first generate words within one subcategory and when word production within this subcategory is exhausted they switch to another subcategory (e.g. Bousfield & Sedgewick, 1944; Gruenewald & Lockhead, 1980; Troyer et al., 1997). It has been suggested that switching is more important for successful performance in phonemic fluency, whereas both strategies seem to be comparably important for semantic fluency (Troyer et al., 1997; Troyer, 2000). Clustering is described as primarily dependent on semantic memory access, whereas switching mainly engages strategic search processes, such as initiation and cognitive flexibility and is decreased under conditions of divided attention (Tröster et al., 1998; Troyer et al., 1997; Troyer, 2000). Accordingly, temporal lobe functioning seems to be crucial for the successful initiation of clustering, whereas the switching strategy seems to rely on fronto-executive functioning and is impaired in patients with frontal lobe lesions or decreased frontal lobe functioning (Haugrud, Crossley, & Vrbancic, 2011; Hirshorn & Thompson-Schill, 2006; Ho et al., 2002; Okruszek, Rutkowska, & Wilińska, 2013; Tröster et al., 1998; Tröster, Woods, Fields, Hanisch, & Beatty, 2002; Troyer et al., 1997, Troyer, Moscovitch, Winocur, Alexander, & Stuss, 1998; Troyer, Moscovitch, Winocur, Alexander, & Stuss, 1998; Weakley & Schmitter-Edgecombe, 2014; Zhao, Guo, & Hong, 2013). Particularly, the left IFG has been identified as relevant for switching during verbal fluency (Hirshorn & Thompson-Schill, 2006). Some studies further emphasize the role of the (superior) parietal cortex for switching in verbal fluency tasks (Gurd et al., 2002; Hirshorn & Thompson-Schill, 2006).

Several studies indicate that, apart from age and education level (Capitani, Laiacona, & Barbarotto, 1999; Tombaugh, Kozak, & Rees, 1999; Troyer et al., 1997; Troyer, 2000), sex affects verbal fluency performance, with women outperforming men (e.g. Burton, Henninger, & Hafetz, 2005, Capitani, Laiacona, & Basso, 1998, 1999, 2005; Filippetti & Allegri, 2011; Halari et al., 2006; Herlitz, Airaksinen, & Nordström, 1999; Hyde & Linn, 1988; Munro et al., 2012; Wallentin, 2009; Weiss, Kemmler, Deisenhammer, Fleischhacker, & Delazer, 2003; Weiss et al., 2006). A robust female advantage has been primarily observed during phonemic fluency, but sex differences in semantic fluency have also been reported (Capitani et al., 1999, 2005; Filippetti & Allegri, 2011; Munro et al., 2012; Scheuringer & Pletzer, 2017; Scheuringer, Wittig, & Pletzer, 2017). While some studies suggest that there are no or only marginal sex differences (Lewin, Wolgers, & Herlitz, 2001; Tombaugh et al., 1999; Troyer, 2000; Weiss, Kemmler, & et al., 2003), verbal fluency seems to be one of the cognitive measures, in which sex differences are most evident (e.g.: Hyde & Linn, 1988; Kimura, 1992; for a review see Andreano & Cahill, 2009). Inconsistencies across studies may be attributable to variations in age range or education level, which both heavily affect verbal fluency performance (Capitani et al., 1999; Tombaugh et al., 1999; Troyer et al., 1997; Troyer, 2000) and interact with sex differences (e.g. Capitani, Laiacona, & Basso, 1998). Furthermore, almost no study on sex differences in verbal fluency did control for women’s hormonal status, i.e. menstrual cycle phase or hormonal contraceptive use, although both have been discussed to affect verbal abilities to a certain extent (e.g. Hampson, 1990; Griksiene & Ruksenas, 2011). Finally, verbal fluency performance and sex differences therein appear to be sensitive to variations in instructions as different wordings may emphasize the use of one strategy over the other (Scheuringer et al., 2017).

Accordingly, it has been suggested that men and women differ in the use of verbal fluency strategies, potentially explaining a female advantage by a more advantageous strategy use in women (Lanting, Haugrud, & Crossley, 2009; Scheuringer & Pletzer, 2017; Weiss et al., 2006). There is some evidence that women switch more often between categories, whereas men generate broader clusters than women (Lanting et al., 2009; Scheuringer & Pletzer, 2017; Weiss et al., 2006). While some studies failed to demonstrate sex differences in strategy use (Brucki & Rocha, 2004; Troyer et al., 1997; Troyer, 2000), a large-scale study by Lanting et al. (2009) indicates that sex differences in strategy use during verbal fluency are stable over a wide age range.

Relatedly, sex differences in the brain correlates of language processing are frequently discussed, and the interest in differences between men and women is still high (for reviews see Kaiser, Haller, Schmitz, & Nitsch, 2009; Wallentin, 2009). Language processing is known to be left hemisphere dominant (e.g. Gur et al., 2000; Hellige, 1993; Knecht et al., 2000; McGlone, 1980; Pujol, Deus, Losilla, & Capdevila, 1999). Early investigations propose that this left-lateralization in the processing of language is stronger in men compared to women (e.g. Baxter et al., 2003; Clements et al., 2006; Jaeger et al., 1998; Kansaku & Kitazawa, 2001; McGlone, 1980; Pugh et al., 1996; Shaywitz et al., 1995; but see Frost, 1999; Weiss, Siedentopf, & et al., 2003; Sommer, Aleman, Bouma, & Kahn, 2004). These differences in lateralization have been attributed to an increased cooperation between the hemispheres in women due to sex hormone actions on inter-hemispheric connectivity along the female menstrual cycle (Bayer, Kessler, Güntürkün, & Hausmann, 2008; Hausmann & Güntürkün, 2000; Hausmann et al., 2002, 2013; Heister, Landis, Regard, & Schroeder-Heister, 1989).

Beside potential differences between men and women in the functional lateralization of language, several neuroimaging studies propose sex differences in regional or whole brain activation within hemispheres during the completion of language tasks (Baxter et al., 2003; Bell, Willson, Wilman, Dave, & Silverstone, 2006; Clements et al., 2006; Gauthier et al., 2009; Konrad et al., 2008; Pugh et al., 1996; Ragland, Coleman, Gur, Glahn, & Gur, 2000; Rossell, Bullmore, Williams, & David, 2002). Only few studies investigate sex differences in brain activation during verbal fluency (Bell et al., 2006; Gauthier et al., 2009; Halari et al., 2006; Schlösser et al., 1998; Soleman et al., 2013; Weiss, Siedentopf, & et al., 2003) or other word-generation tasks (Gizewski, Krause, Wanke, Forsting, & Senf, 2006; Konrad et al., 2008). While some of these studies observe similar brain activation in men and women (Schlösser et al., 1998; Weiss, Siedentopf, & et al., 2003), the majority report stronger activation of language areas in men compared to women (Bell et al., 2006; Gauthier et al., 2009; Gizewski et al., 2006; Halari et al., 2006; Konrad et al., 2008; Soleman et al., 2013). For instance, Bell et al. (2006) reported that men, compared to women showed stronger activation in the right and left DLPFC, the right inferior parietal gyrus and cingulate during the completion of a phonemic fluency task. In a study by Gauthier et al. (2009) men showed stronger activation in the left inferior temporal gyrus, the cerebellum, the anterior and posterior cingulate cortices and right frontal areas. Furthermore, it has been suggested that levels of performance affect sex differences in brain activation (Gauthier et al., 2009; Weiss, Siedentopf, & et al., 2003). Weiss, Siedentopf, & et al. (2003) reported that no or only marginal sex differences in brain activation are apparent when testing only high performers. Most of these studies use a phonemic fluency task to assess sex differences (Bell et al., 2006; Gauthier et al., 2009; Halari et al., 2006; Schlösser et al., 1998; Weiss, Siedentopf, & et al., 2003). Only one study focusing on transsexual adolescents has used a semantic fluency task (Soleman et al., 2013). They found stronger activation in the rolandic operculum in boys compared to girls (Soleman et al., 2013).

One potential reason for inconsistent findings regarding sex differences in brain activation during verbal fluency is a lack of control for the female menstrual cycle. Some studies indicate that sex steroid fluctuations along the menstrual cycle influence brain activation during cognitive tasks (Dietrich et al., 2001; Fernández et al., 2003; Konrad et al., 2008; Sommer, Aleman, Somers, Boks, & Kahn, 2008), including verbal fluency (Pletzer, Harris, Scheuringer, & Hidalgo-Lopez, 2019). Specifically, the lateralization of language functions has been demonstrated to fluctuate along the menstrual cycle (Hausmann & Güntürkün, 2002; Hausmann, Becker, Gather, & Güntürkün, 2002; Hjelmervik et al., 2012; Weis et al., 2008). In a recent study, we observed stronger activation in the left hippocampus during the pre-ovulatory phase of the menstrual cycle, when estradiol levels peak, and stronger activation in right fronto-striatal areas during the luteal phase of the menstrual cycle, when progesterone levels peak (Pletzer et al., 2019). Furthermore, activation in the bilateral IFG was stronger during the luteal phase of the menstrual cycle. It is thus possible, that the strength of sex differences depends on women’s cycle phase and that some sex differences emerge only in certain cycle phases. Note however, that these results were of moderate effect size and emerged in ROI-based analyses, while no menstrual cycle effects emerged at the whole-brain level (Pletzer et al., 2019).

Furthermore, none of the previous studies on sex differences in brain activation during verbal fluency has controlled for the use of different strategies in men and women. So far, no study has addressed, whether sex differences in brain activation during verbal fluency vary depending on whether a clustering or switching strategy is used. If sex differences in brain activation patterns during verbal fluency are – as has sometimes been suggested – reflective of different strategy use in women, these sex differences should disappear, if men and women are required to use the same strategy. If however, sex differences in brain activation during verbal fluency are reflective of additional effort employed in men to uphold task performance, sex differences should persist, if men and women are required to use the same strategy. It is even possible, that instructing strategy increases sex differences in brain activation, given the assumption that men and women prefer different strategies during verbal fluency. Accordingly, additional effort should be required, when instructed to use the non-preferred strategy.

To address the issue of strategy-dependent sex differences during semantic verbal fluency, we recruited a sample of 36 healthy young men to perform the same verbal fluency task during fMRI as the sample of 36 healthy young women, in which we previously addressed menstrual cycle changes in verbal fluency (Pletzer et al., 2019). This allows us to control for variations in the size of sex differences depending on the female menstrual cycle and address sex hormone levels as potential moderators of sex differences in brain activation during verbal fluency. Importantly, no strategy-dependent shifts in brain activation were observed along the menstrual cycle (Pletzer et al., 2019), suggesting that, while overall activation patterns are cycle-dependent, strategy-dependent activation is not. The male sample was tightly matched to the female sample in age, IQ and education. Since women of the female sample were tested three times along the menstrual cycle, men were also tested thrice to control for potential learning effects on the task. In a first step we explore the different brain networks supporting clustering and switching during semantic verbal fluency, since this has not been previously addressed in an adult population. We hypothesize stronger activation in temporal areas for clustering, and stronger activation in fronto-parietal areas during switching. In a second step, we address sex differences in these networks. Building on previous studies, we hypothesize stronger activation in the overall verbal fluency network, particularly the left IFG and stronger lateralization of brain activation during verbal fluency, for men compared to women. We further explore, how sex differences are modulated by verbal fluency strategy.

## 2 Methods

### 2.1 Participants

A total of 72 healthy, right-handed German native-speakers (36 men, 36 women), aged between 20 and 34 years were included in this study. They reported no neurological, endocrine and mental disorders and did not use hormonal contraception or medication. Two participants whose responses indicated a lack of compliance to the task instructions were excluded from all analyses, resulting in a final sample of 35 men and 35 women. Mean age of participants did not differ significantly (*t*_(68)_ = 0.53, *p* = .59) between men (*M* = 24.66; SD = 3.66) and women (*M* = 25.17; *SD* = 4.28). Participants were recruited via advertisements in newspapers, at the university, and in social media. All participants had a minimum of nine years of education, and most participants had either passed general qualification for university entrance (*n* = 32, 45.7%) or had a university degree (*n* = 24; 34.3%). The average IQ of participants as assessed with the screening version of the Advanced Progressive Matrices (APM; Raven, Raven, & John Hugh Court, 1962) ranged between 82 and 120, and did not differ significantly (*t*_(66)_ = 1.29, *p* = .20) between men (*M* = 107.52, *SD* = 11.01) and women (*M* = 110.77, SD = 9.61). Menstrual cycle length of women ranged from 25 to 35 days (*M* = 28.80; SD = 2.61).

### 2.2 Ethics statement

All participants gave written informed consent to participate in the study. The study procedures were in accordance with the Code of Ethics of the World Medical Association (Declaration of Helsinki). The experiment was approved by the University of Salzburg’s ethics committee.

### 2.3 Procedure

The verbal fluency task was part of a larger study aiming to investigate sex- and menstrual cycle effects in brain activation during the use of different cognitive strategies. To account for possible effects of the female menstrual cycle and balance potential learning effects between the sexes, all participants were tested three times. In women, scanning sessions were time-locked to menses, pre-ovulatory phase and mid-luteal phase. Details on menstrual cycle staging and menstrual cycle effects in the female sample have already been described in Pletzer et al. (2019). Importantly, no menstrual cycle effects were observed at the whole-brain level and in regions of interest, the strength of sex differences varies across cycle phases (compare Statistical analyses).

Each scanning session started with an eight minute resting state scan, followed by a 35 min task-based functional scan. During this scan, participants completed 20 blocks of verbal fluency, ten per condition (i.e. clustering and switching). Verbal fluency blocks lasted for 30 s and were alternated with spatial navigation blocks (also 30 s per block), separated by a 15-second inter-stimulus interval, which also lasted 30 s per items. The first block of the task-based scan was always a navigation block. Results on the navigation task are reported elsewhere (Harris et al., in prep). Among the verbal fluency blocks, clustering and switching instructions were alternated (compare Verbal Fluency Task). Three task versions were generated using the Unreal Engine 4, Version 1. Order of task versions was counterbalanced across scanning sessions and cycle phases. After the task-based scan, a high resolution structural scan and a DTI scan were completed, during which participants watched a movie. In total, one scanning session took around 70 min to complete.

### 2.4 Verbal fluency task

The semantic fluency task used in the present fMRI-study was validated on a large sample (30 men, 30 women) beforehand (compare Supplementary Material 1). During each verbal fluency block, a semantic category (e.g. ‘fruits’) was presented on an MR-compatible screen for 30 s. Per task version, 20 different categories were used. All categories of the verbal fluency task were presented in German. Participant’s task was to generate as many words as possible belonging to this category, within 30 s. To avoid motion artifacts during scanning, participants were instructed to generate words silently (i.e. covert verbal fluency task).

To modulate the verbal fluency strategies used by the participants, two different instructions were employed: the clustering and the switching instruction. Instructions were indicated via a cue above the category presented on the screen. During the clustering condition, participants were asked to make sure that consecutive words generated belonged to the same semantic subcategory and to switch subcategories only, if no further words belonging to a subcategory came to mind. For example, the words produced for the semantic category ‘sports’ might be clustered according to ‘aquatic sports’, ‘winter sports’, ‘ball sports’ and so on (e.g. swimming, surfing, sailing, …., skiing, snow-boarding, ice-scating, …, tennis, volley-ball, basket-ball, …). Importantly, subcategories were not given, so participants were free to construct them themselves. During the switching condition participants were asked to make sure that consecutively generated words did not belong to the same subcategory (e.g.: swimming, skiing, tennis, surfing, volley-ball, sailing, snow-boarding, basket-ball, ice-scating, …). Within and across task versions clustering and switching condition were matched (compare Supplementary Material 1) for overall difficulty (number of words generated during neutral instructions), clustering difficulty (number of words generated during clustering instructions) and switching difficulty (number of words generated during switching instructions).

To obtain at a rough estimate of performance during silent word generation, participants were asked to press one of two possible buttons whenever a word came to mind. When switching to another subcategory, participants should switch to the other button. Accordingly, participants pressed the same button for each word within the same subcategory, and switched to another button when switching to another subcategory. The number and speed of button presses was evaluated to obtain an estimate of the number of words produced and the corresponding reaction time. Furthermore, the number of switches and the average cluster size was evaluated in the clustering condition. From these analyses we excluded responses of six participants (for two no responses were recorded in at least one session, two used button presses to indicate switches rather than words and for two buttons presses were recorded in duplicate). In contrast, the two participants that were excluded from all analyses showed clear switching behavior during the clustering condition or vice versa, indicating that they did not differentiate between the two types of instructions.

### 2.5 Hormone analyses

For hormone analyses, participants gave three saliva samples: one upon arrival at the lab after rinsing their mouth, one immediately before entering the scanner and one after the scanning session. Prior to hormone assessment, saliva samples were stored at −20 °C and centrifuged twice at 3000 rpm for 15 and 10 min, respectively. Estradiol was assessed using the HS Estradiol in Saliva ELISA by Salimetrics, while progesterone and testosterone were assessed for each sample using salivary ELISA kits by DeMediTec. As recommended by the kit instructions, for each participant and test session, the three saliva samples were pooled prior to analysis in order to control for fluctuations in hormone and saliva production and obtain a more stable measure of the circulating hormone levels during the scanning session. All samples were assessed in duplicates and analysis of samples with > 25% variation between duplicate measures was repeated.

### 2.6 MRI data acquisition

A Siemens Magnetom Trio Tim 3 Tesla scanner, located at the Christian Doppler Klinik (Salzburg, Austria), was used to acquire whole-brain fMRI data. For the task-based functional scan, a T2^*^-weighted gradient echo planar (EPI) sequence sensitive to BOLD contrast was used (TR = 2250 ms, TE = 30 ms, FOV 192 mm, matrix size 192x192, slice thickness = 3.0 mm, flip angle 70°, voxel size 3.0 × 3.0 × 3.0 mm, 36 transversal slices parallel to the AC-PC line). High-resolution structural images were acquired using a T1-weighted sagittal 3D MPRAGE sequence (TR = 2300 ms, TE = 2.91 ms, TI delay of 900 ms, FOV 256 mm, slice thickness = 1.00 mm, flip angle 9°, voxel size 1.0 × 1.0 × 1.0 mm, 160 sagittal slices). Resting state and DTI data are not described in the present manuscript.

### 2.7 MRI data analysis

MRI data analysis was performed as also described for menstrual cycle effects in Pletzer et al. (2019). Prior to analysis, the first 6 images of each session were discarded. As a first pre-processing step, images were despiked using the 3d-despiking procedure as implemented in AFNI (afni.nimh.nih.gov). SPM12 standard procedures and templates were used for further preprocessing, including realignment of functional images, slice-timing, segmentation and normalization of structural images using the computational anatomy toolbox (CAT12), normalization of functional images using the normalization parameters obtained by CAT12, smoothing of normalized images using a 6 mm Gaussian kernel and resampling to 3x3x3 isotropic voxels. Additionally, after the realignment step, physiological noise was identified using a biophysically-based model (Tierney et al., 2016). Via the Functional Image Artefact Correction Heuristic (FIACH, Tierney et al., 2016), images were filtered and 6 regressors of physiological noise were extracted.

After preprocessing, a 2-stage mixed effects model was applied. In the subject-dependent fixed-effects first-level analysis, we modelled one regressor per verbal fluency category (clustering and switching) for each session of each subject, by convolving the duration of the event with the canonical hemodynamic response function implemented in SPM12. As regressors of no interest, instructions and navigation trials were modelled separately, as were the 6 realignment parameters and 6 physiological noise parameters obtained from the FIACH procedure. Autocorrelation correction was performed using an AR(1) model (Friston et al., 2002) and a high pass filter cut-off was set at 128 s. For each of the two regressors of interest, one statistical contrast was defined to compare BOLD-response during each category to baseline, resulting in two contrast images (activation maps) for clustering and switching respectively for each session of each subject, i.e. six contrast images per subject.

After that, the analysis approach was two-fold. In a first-step, region-of interest (ROI)-based analyses were performed by extracting principle eigenvariates as measures of BOLD-response from a one-sample T-test second-level design including all first-level contrast images. ROIs included well-described language areas, i.e. the left and right IFG (Broca’s area, BA 44/45) as well as the left superior temporal gyrus (Wernicke’s area, BA 22). ROIs were defined using the Wake Forest University (WFU) Pickatlas toolbox (Maldjian, Laurienti, Kraft, & Burdette, 2003). Eigenvalues were compared between sexes and condition using linear mixed effects models (compare Statistical analysis section). Although not the focus of the present manuscript, for comparison to previous menstrual cycle analyses, eigenvalues extracted from the left and right hippocampus, the left and right caudate, as well as the left and right DLPFC (BA 46), were also compared between men and women (compare Supplementary Material 2).

In a second step, differences in brain activation due to sex or condition were explored at the whole brain level. Contrast images (activation maps) were entered into a flexible factorial design modeling the following factors: (i) *subject*, to control for within-subject variation (independence: yes, variance: equal), (ii) *between-subject factor/group*, i.e. sex (independence: yes, variance: unequal), and (iii) *within-subject factor/condition*, i.e. instruction^*^cycle phase (independence: no, variance: equal). The interaction between factors (ii) and (iii) was also modelled in order to be able to address interactions between sex and condition and their modulation by cycle phase. In particular, we wanted to assess if sex differences were moderated by menstrual cycle phase in women. Please note that the results were not altered by removing cycle phase and all respective interactions from the models. Session was entered as covariate to control for learning effects.

Even though no whole-brain menstrual-cycle effects were previously observed in the female sample (Pletzer et al., 2019), menstrual cycle phase was controlled in the whole brain analyses, since also nonsignificant variations along the menstrual cycle could affect the strength of the observed sex differences. To that end, the three sessions of men were reordered to make sure, that the sex*cycle interaction was not confounded by learning effects, since some women had their phase 1 scan in the first session, some in the second and some in the third session. In order to achieve that, men’s sessions were randomly assigned a virtual cycle phase in such a way that virtual cycle phases in men were counterbalanced across scanning sessions in the same way that actual cycle phases were counterbalanced across scanning sessions in women. Accordingly, the same number of men and women had phase 1 assigned to their first, second or third scanning session, in women corresponding to their hormonal status, in men irrespective thereof.

The following F-contrasts were defined as described by Glaescher and Gitelman (2008): main effect of cycle in women, main effect of (virtual) cycle in men, sex*cycle interaction, main effect of sex, condition*cycle interaction, main effect of condition, sex*cycle*condition interaction, sex*condition interaction, main effect of session. If F-contrasts revealed significant clusters, separate T-contrasts for positive and negative effects were defined to clarify the directionality of effects and results of these T-contrasts are reported in the results section.

To address, whether sex differences were mediated via sex hormone influences, additional flexible factorial designs were created, using z-standardized estradiol, progesterone and testosterone values as additional covariate and modelling their interaction with sex. The following F-contrasts were defined: *main effect of hormone across all participants, main effect of hormone in women*, *main effect of hormone in men*, sex*-*hormone interaction*. If F-contrasts revealed significant clusters, separate T-contrasts for positive and negative effects were defined to clarify the directionality of effects and results of these T-contrasts are reported in the results section.

For all second-level designs, we used an extent threshold of k = 50 voxels, an uncorrected primary threshold of p < 0.001 and a secondary cluster-level FWE-corrected threshold of p < 0.05 (indicated as p_FWE_). Peak-level FWE-corrected p-values are also reported.

### 2.8 Lateralization indices

To evaluate if the verbal fluency task induces stronger activation in the left hemisphere and if lateralization differs between men and women, we extracted lateralization indices (LI) for the frontal ROIs (IFG) using the LI toolbox for SPM12 (Wilke & Lidzba, 2007). Lateralization indices range from −1 to 1 and represent the extent to which activation is stronger in one hemisphere compared to the other. Positive values represent left-lateralization and negative values representing right lateralization (Wilke & Lidzba, 2007).

### 2.9 Connectivity analyses

Connectivity analyses using left and right IFG (Broca’s area), as well as the left STG (Wernicke’s area) as seeds were performed using the CONN-toolbox (Whitfield-Gabrieli & Nieto-Castanon, 2012). The pre-processed functional images underwent linear detrending for white matter (WM) and cerebrospinal fluid (CSF) influences, a band-pass filter (0.008–0.09 Hz) and motion-correction. ROI-to-voxel connectivity analyses were used to create voxel-wise connectivity maps for each subject and session. Connectivity maps were then subjected to the same flexible factorial design as activation maps modeling the factors subject, sex and condition (instruction/cycle phase) as well as the sex*condition interaction and entering session as a covariate. Again, it was addressed whether sex differences were mediated via sex hormone influences, by creating additional flexible factorial designs using z-standardized estradiol, progesterone and testosterone values, respectively, as additional covariates and modelling their interaction with sex. In all models contrasts were defined as for activation results. Like for activation, we used an extent threshold of k = 50 voxels, an uncorrected primary threshold of p < 0.001 and a secondary cluster-level FWE-corrected threshold of p < 0.05 (indicated as p_FWE_). Peak-level FWE-corrected p-values are also reported.

### 2.10 Statistical analyses

Statistical analysis was carried out using R 3.3.0. Hormone values, performance, brain activation in specified ROIs and lateralization indices were compared between men and women in the context of linear mixed effects models using the lme function of the nlme package (Pinheiro, Bates, DebRoy, Sarkar, & Team, 2013). All models included participant number (PNr) as a random factor and session as a fixed effect. Effects of test session are reported in Supplementary Material 3. Models for performance, brain activation and lateralization indices additionally included instruction as a fixed effect. To assess sex differences, we followed the following rationale:

In a first step, it was assessed, whether sex differences varied along the menstrual cycle, by including the interaction term sex*cycle in the model [e.g.: IFG ~ session + instruction*sex*cycle + (1|PNr)]. For the factor cycle, menses was used as reference category. If a significant sex*cycle interaction was observed (as was the case for hormone values), post-hoc analyses were performed, comparing men and women separately for each cycle phase. Results of these post-hoc analyses are reported and were FDR-corrected over three comparisons. If no significant sex*cycle interaction was observed (as was the case for all other measures), cycle phase was dropped from the model [e.g.: IFG ~ session + instruction*sex + (1|PNr)] and results of the model without cycle phase are reported, FDR-corrected for the number of comparisons (4 performance measures, 3 regions of interest).

If a significant sex difference was observed, it was tested in a third step, whether this sex difference was moderated via sex hormones. To that end, hormone levels were added to the model [e.g. LI ~ 1|PNr + session + instruction*sex*hormone + (1|PNr)]. Since three hormones were tested, p-values for these hormonal analyses were FDR-corrected over the three analyses. All continuous dependent and independent variables were z-standardized prior to analyses, such that the coefficient b of fixed effects in the models represents a standardized effect size based on standard deviations, similar to Cohen’s d.

### 2.11 Data availability

Data and scripts for ROI-analyses are openly available at http://webapps.ccns.sbg.ac.at/OpenData/. MR-images for whole-brain analyses are available from the corresponding author upon reasonable request.

## 3 Results

### 3.1 Hormone results

Analyses of estradiol and progesterone revealed significant sex*-cycle interactions (both |b| > 0.88, both *SE*_b_ < 0.27; both |*t*|> 3.63, both *p* < .001). Women had significantly higher estradiol levels than men only during their pre-ovulatory phase (*t*_(50.99)_ = 2.62, p_FDR_ = 0.03, d = 0.63), but not during menses and luteal phase (both |*t*| < 0.72, both *p* > .47). Women had significantly higher progesterone levels than men only during their luteal phase (*t*_(63.76)_ = 3.00, *p*_FDR_ = 0.01, d = 0.78) but not during menses and pre-ovulatory phase (both |*t*| < 1.75, both *p* > .08). Irrespective of cycle phase, testosterone was significantly higher in men compared to women (*b* = 1.33, *SE*_b_ = 0.16; *t*_(68)_ = 8.06, *p* < .001, Table 1).

### 3.2 Behavioral results

None of the behavioral parameters were affected by menstrual cycle phase or interactions between cycle phase and other factors (all |*b*| < 0.15, all |*t*| < 1.20, all *p* > 0.23). Accordingly, cycle phase was dropped from the models. Participants produced more words, faster reactions, larger clusters and fewer switches under the clustering, compared to the switching instruction (all |*b*| > 0.30, all *SE*_b_ < 0.09, all |*t*_(3 3 1)_| > 3.57, all *p*_FDR_ < 0.01). Sex did not affect any of the behavioral parameters and did not interact with instruction for any of the behavioral parameters (all |*b*| < 0.12, all *SE*_b_ > 0.07, all |*t*| < 1.11, all *p* > .27; compare Table 2).

### 3.3 ROI-based analyses

**Fig. 1:**
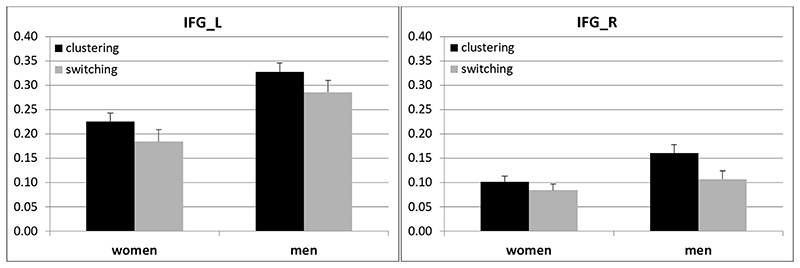
BOLD-responses in the left IFG (left panel) and the right IFG (right panel), separately for the clustering and switching instruction in women and men. Error bars represent standard errors.

In none of the ROIs did we observe a significant interaction between cycle phase and any other factor (all |*b*| < 0.24, all |*t*| < 0.48, all *p* > 0.14). Accordingly, cycle phase was dropped from the models. BOLD-response in the left IFG (Fig. 1, left panel) was stronger under the clustering (*M* = 0.27, *SE* = 0.02), compared to the switching instruction (*M* = 0.23, *SE* = 0.02; *b* = – 0.18; *SE*_b_ = 0.08, *t*_(3 4 7)_ = −2.35, *p* = .02). Furthermore, BOLD-response in the left IFG was stronger in men (*M* = 0.30, *SE* = 0.03) compared to women (*M* = 0.20 *SE* = 0.03; *b* = 0.45, *SE*_b_ = 0.21, *t*_(68)_ = 2.16, *p* = .03). There was no significant interaction between sex and instruction (*b* = −0.002, *SE*_b_ = 0.11, *t*_(3 4 7)_ = −0.02, *p* = .99). Sex hormones did not affect BOLD-response in the left IFG (all |*b*| < 0.12, all |*t*| < 0.95, all *p* > 0.34). For activation in the right IFG/the right Broca’s area (Fig. 1, right panel), as well as the left STG/Wernicke’s area, we did not observe significant effects of sex or instruction (all |*b*| < 0.41, all |*t*| < 1.97, all *p* > .05).

### 3.4 Whole-brain analyses

Overall, the verbal fluency task activated a broad bilateral network, extending from frontal over temporal-parietal to occipital areas. Deactivation was observed in typical default mode areas, including cingulate cortex, pre- and paracentral areas, extending to the medial temporal lobe.

#### 3.4.1 Main effect of instruction

**Fig. 2:**
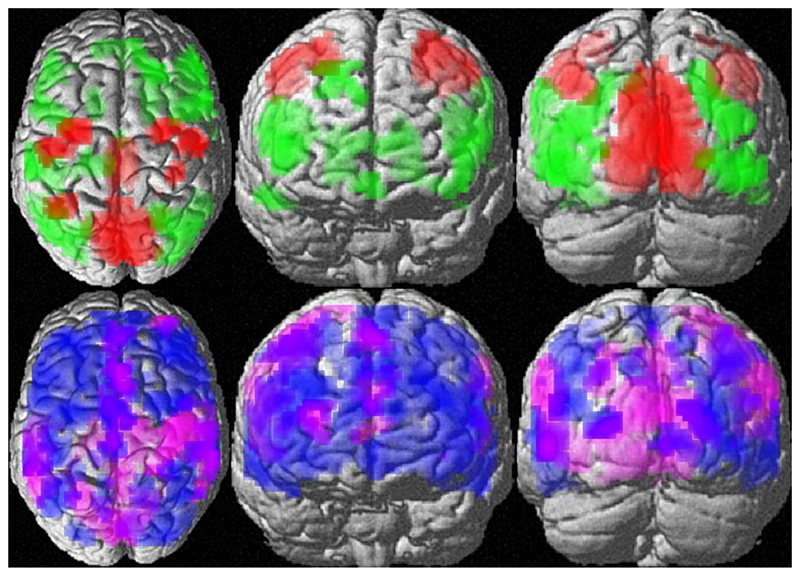
Effects of instruction (upper panel) and sex (lower panel) on whole-brain activation patterns. Upper panel: Areas of stronger activation during clustering compared to the switching are displayed in green. Areas of stronger activation in the switching compared to the clustering condition are displayed in red. Lower panel: Areas of stronger activation in men compared to women are shown in blue. Areas of stronger activation in women compared to men are displayed in magenta. Areas of stronger activation in men overlap areas of stronger activation during clustering. Areas of stronger activation in women overlap areas of stronger activation during switching.

Over all participants, the clustering instruction resulted in stronger activation compared to the switching instruction in a large network of mainly occipito-temporal and frontal areas (Suppl.4 Table 1, Fig. 2, upper panel, green). Stronger BOLD-response under the switching compared to the clustering instruction was observed in the posterior/middle cingulate gyrus and medial occipital areas (less deactivation), as well as bilateral superior parietal and central areas (more activation; Suppl.4 Table 1, Fig. 2, upper panel, red).

#### 3.4.2 Main effect of sex

Irrespective of instruction and menstrual cycle phase, we observed stronger BOLD-response in women compared to men in the posterior/ middle cingulate and medial occipital gyri (less deactivation), as well as in right central gyri (more activation; Suppl.4 Table 2, Fig. 2, lower panel magenta). Stronger activation in men, compared to women was found in an extended bilateral fronto-temporal network (Suppl.4 Table 2, Fig. 2, lower panel blue).

#### 3.4.3 Interactive effects of sex and instruction

**Fig. 3:**
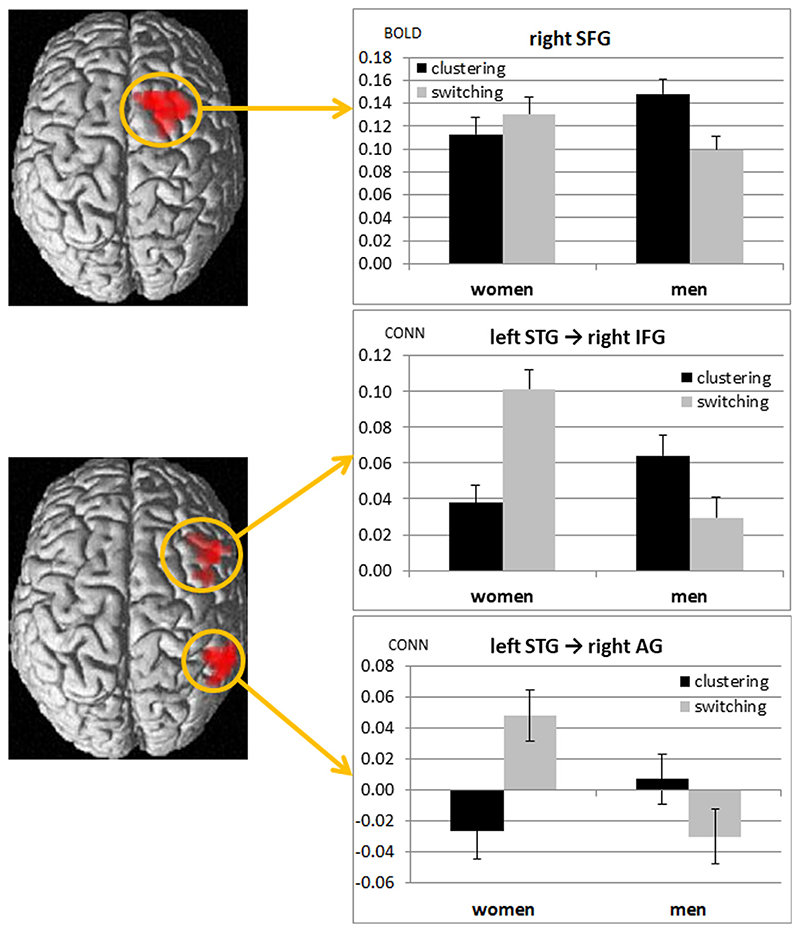
Interactive effect of sex and instruction in activation of the right superior frontal gyrus (upper panel) and connectivity of Wernicke’s area (lower panel). Upper panel: In women, stronger activation in the right superior frontal gyrus (SFG) was observed during switching compared to clustering. In men, stronger activation was observed during clustering compared to switching. Lower panel: Inter-hemispheric connectivity is increased during switching compared to clustering in women, but during clustering compared to switching in men. Error bars represent standard errors.

An interactive effect of sex and instruction was found irrespective of menstrual cycle phase in the right superior frontal gyrus ([21, 11, 43], *T* = 4.62, k = 167 voxels, cluster *p*_FWE_ < 0.001, peak *p*_FWE_ = 0.049; Fig. 3, upper panel), where stronger activation in women was observed under the switching compared to the clustering instruction, whereas the opposite was true for men.

#### 3.4.4 Effects of sex hormones

Estradiol and progesterone did not affect brain activation in either men or women. In men, but not in women, testosterone was significantly positively related to activation in the left parietal operculum ([–57, 34, 22], k = 133 voxels, T = 5.92, cluster p_FWE_ = 0.001, peak p_FWE_ < 0.001), left middle temporal gyrus ([–60, −16, −11], k = 59 voxels, T = 5.68, cluster p_FWE_ = 0.04, peak p_FWE_ < 0.001) and right middle frontal gyrus ([39, 29, 16], k = 91 voxels, T = 4.61, cluster p_FWE_ = 0.007, peak p_FWE_ = 0.05).

### 3.5 Lateralization indices

**Fig. 4:**
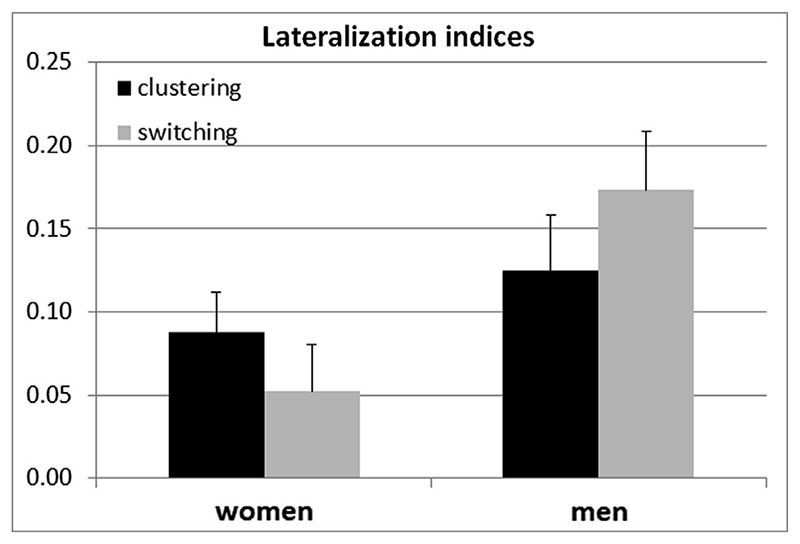
Lateralization indices in the IFG (left panel) and and right IFG (right panel). Left-lateralization is reduced during switching compared to clustering in women, but during clustering compared to switching in men. Error bars represent standard errors.

Overall, lateralization indices in the IFG were positive (women: LI = 0.07, *SD* = 0.26; men: LI = 0.15, *SD* = 0.35), indicating that activation in this area was primarily left-lateralized (compare also Fig. 1). Lateralization indices were not affected by menstrual cycle phase or interactions between cycle phase and other factors (all |*b*| < 0.40, all |*t*| < 1.37, all *p* > 0.18). Accordingly, cycle phase was dropped from the model. We observed no significant main effects of sex or instruction (both |*b*| < 0.12, both |*t*| < 1.40, both *p* > 0.17), but a significant interaction between sex and instruction (*b* = 0.26, *SE*_b_ = 0.12, *t*_(3 4 6)_ = 2.26, *p* = 0.02). Men showed stronger left-lateralization compared to women, specifically under the switching instruction (Fig. 4). In women, activation was more left-lateralized during clustering compared to switching, while in men activation was more left-lateralized during switching compared to clustering. There were no significant effects of sex hormones on lateralization (all |*b*| < 0.22, all |*t*| < 1.72, all p > .08).

### 3.6 Connectivity analyses

#### 3.6.1 Main effect of instruction

During clustering, the left and right IFG showed significantly stronger connectivity to the right middle occipital gyrus (left: [33, −85, 16], *T* = 4.04, k = 79 voxels, cluster p_FWE_ < 0.001, peak p_FWE_ = 0.86; right: ([15, −82, 7], T = 4.64, k = 260 voxels, cluster p_FWE_ < 0.001, peak p_FWE_ = 0.17). During switching, the left IFG, but not the right IFG, showed significantly stronger connectivity to the right angular gyrus ([45, −37, 55], *T* = 4.40, k = 103 voxels, cluster *p*FWE < 0.001, peak *p*_FWE_ = 0.42) and the left precentral gyrus ([−54, −19, 46], *T* = 4.62, k = 236 voxels, cluster *p*FWE < 0.001, peak *p*_FWE_ = 0.18).

The left STG (Wernicke’s area) showed significantly stronger connectivity to the left superior parietal lobe during switching compared to clustering ([−27, −49, 64], *T* = 4.62, k = 124 voxels, cluster *p*_FWE_ < 0.001, peak *p*_FWE_ = 0.188) and left precentral gyrus ([−30, −7, 61], *T* = 4.45, k = 54 voxels, cluster *p*_FWE_ = 0.004, peak *p*_FWE_ = 0.355). No area showed stronger connectivity to the left STG during clustering.

#### 3.6.2 Main effect of sex

**Fig. 5:**
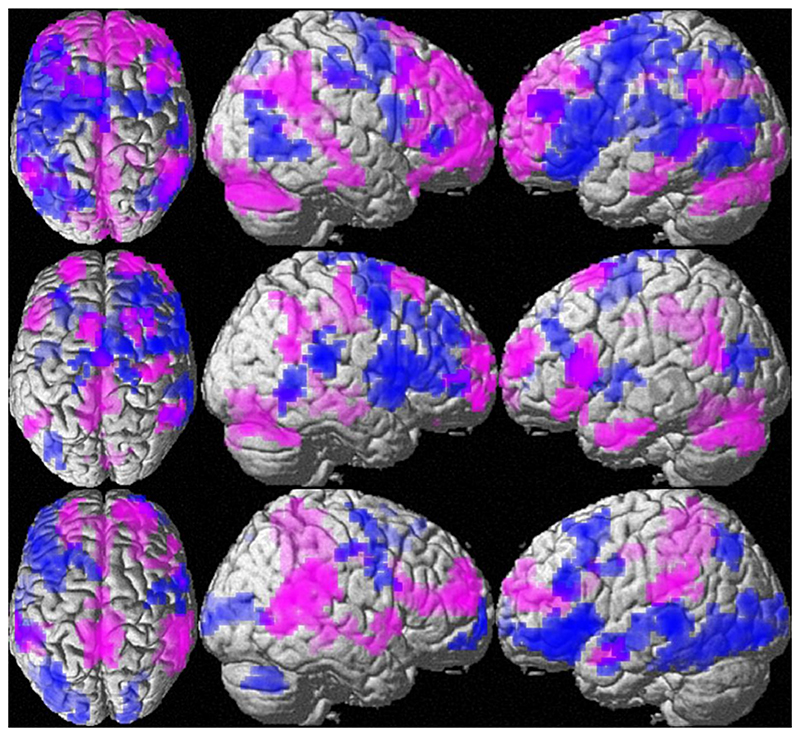
Sex differences in connectivity of the left IFG (upper panel), right IFG (middle panel), left STG (lower panel). Areas of stronger connectivity in men compared to women are displayed in blue. Areas of stronger connectivity in women compared to men are displayed in magenta. For all seeds, connectivity was stronger to ipsi-lateral areas in men, but contra-lateral areas in women. IFG = inferior frontal gyrus (Broca’s area), STG = superior temporal gyrus (Wernicke’s area).

Irrespective of instruction and menstrual cycle phase, women showed stronger connectivity than men from both, the left IFG (Suppl.4 Table 3, Fig. 5, upper panel) and right IFG (Suppl.4 Table 4, Fig. 5, middle panel), to bilateral middle frontal gyri, inferior parietal lobules, PCC/precuneus and cerebellum. Men however showed stronger connectivity than women from the left IGT to bilateral inferior frontal, precentral and middle temporal gyri with substantially larger clusters in the left hemisphere and from the right IFG to right hemispheric frontal and temporal areas.

Irrespective of instruction and menstrual cycle phase, women showed stronger connectivity than men from the left posterior STG (Wernicke’s area; Suppl.4 Table 5, Fig. 5, lower panel) to the bilateral superior frontal gyri, inferior parietal lobule, posterior and anterior cingulate cortex. Men showed stronger connectivity than women to left-hemispheric frontal and temporal areas.

#### 3.6.3 Interactive effects of sex and instruction

For connectivity of the left and right IFG no significant interactive effects between sex and condition were observed. Connectivity of the left STG showed a significant interactive effect in the right inferior frontal gyrus ([39 23 10], *T* = 4.28, k = 84 voxels, cluster *p*_FWE_ < 0.001, peak *p*_FWE_ = 0.56) and right angular gyrus ([57–52 40], *T* = 4.60, k = 61 voxels, cluster *p*_FWE_ = 0.002, peak *p*_FWE_ = 0.20). Connectivity to these areas was stronger during switching compared to clustering in women, but to clustering compared to switching in men (Fig. 3, lower panel).

#### 3.6.4 Effects of sex hormones

Connectivity of the left IFG and left STG were not affected by sex hormone levels in either men or women. In men, but not women, both estradiol and testosterone were significantly positively related to connectivity within the right IFG (E: [63, 17, 7], k = 77 voxels, T = 9.86, cluster p_FWE_ < 0.001, peak p_FWE_ < 0.001; T: [60, 23, 16], k = 138 voxels, T = 11.09, cluster pFWE < 0.001, peak p_FWE_ < 0.001). Furthermore, in men, testosterone was significantly positively related to connectivity between the right IFG and right-hemispheric language areas, including the right temporal pole ([48, −10, −38], k = 78 voxels, T = 10.70, cluster p_FWE_ < 0.001, peak p_FWE_ < 0.001), right angular gyrus ([51, −40, 61], k = 81 voxels, T = 7.37, cluster p_FWE_ < 0.001, peak p_FWE_ < 0.001) and right superior frontal gyrus ([9, 65, 28], k = 55 voxels, T = 5.69, cluster p_FWE_ = 0.003, peak p_FWE_ = 0.001). The effect in the right superior frontal gyrus was also confirmed in women ([28, 68, 19], k = 54 voxels, T = 7.88, cluster p_FWE_ = 0.003, peak p_FWE_ < 0.001) and accordingly across all participants ([15, 68, 22], k = 114 voxels, T = 8.24, cluster p_FWE_ < 0.001, peak p_FWE_ < 0.001).

## 4 Discussion

Verbal fluency is supported by two different strategies – clustering and switching. Several neuroimaging and brain lesion studies suggest that clustering and switching recruit partially different brain networks. However, these networks have not been studied in adult populations during semantic fluency. Sex differences in verbal fluency performance are of high interest, with most studies suggesting women to outperform men. Some studies do further suggest sex differences in the use of verbal fluency strategies. However, the neural underpinnings of these sex differences in verbal fluency strategies are unclear. The recent study was designed to contrast brain activation and connectivity during clustering and switching in a semantic fluency task between men and women.

Based on previous findings, we hypothesized (i) stronger activation during clustering compared to switching in temporal areas, (ii) stronger activation during switching compared to clustering in frontal and superior parietal areas, (iii) stronger activation, specifically in frontal areas in men compared to women, as well as (iv) increased left-lateralization in men, compared to women.

Furthermore, we explore the interactive effects of sex and condition on brain activation and lateralization, as well as sex differences in connectivity patterns.

Before entering into the discussion of our results, it is important to note one limitation of the present study. Like many neuroimaging studies on verbal fluency the present study optimized the assessment of brain activation at the expense of precise behavioral measurements. Accordingly, the behavioral measures obtained during the present study are estimates at best and their interpretability is limited. Therefore, we sometimes compare our neuroimaging results to results of previous behavioral studies, but urge the reader to keep in mind that these results have not been confirmed in the present study. Specifically, we did not observe any sex differences in verbal fluency performance in the present study.

On the flipside however, an important strength of the present study is that our sample was strictly controlled for hormonal status, excluding women on hormonal contraceptives and scheduling test sessions in three clearly defined cycle phases. While previous results do suggest menstrual cycle effects in the bilateral IFG (Pletzer et al., 2019), our results indicate that sex differences in the left IFG surpass these cycle effects with stronger activation in men irrespective of women’s cycle phase. The same holds true for whole-brain results regarding sex differences, since no whole-brain menstrual cycle effects were observed in women (Pletzer et al., 2019) and menstrual cycle did not interact with sex in the present study.

Regarding task effects, our results support the notion that clustering is accompanied by stronger recruitment of temporal areas, while switching is accompanied by stronger recruitment of superior parietal areas. However, we could not find support for the notion that the participation of frontal areas is greater during switching, compared to clustering. On the contrary, we even found stronger activation in frontal areas during clustering, compared to switching. One possible explanation for inconsistencies between our results and previous studies (e.g. Hirshorn & Thompson-Schill, 2006; Okruszek et al., 2013; Tröster et al., 2002; Troyer, Moscovitch, Winocur, Alexander, & et al., 1998; Troyer, Moscovitch, Winocur, Leach, & et al., 1998) concerning the relation between the IFG and switching is the differential task design. Previous findings on the dissociation of clustering and switching mainly originate from lesion studies or studies investigating different neurological patient groups (Haugrud et al., 2011; Ho et al., 2002; Okruszek et al., 2013; Tröster et al., 1998, 2002; Troyer, Moscovitch, Winocur, Alexander, & et al., 1998; Troyer, Moscovitch, Winocur, Leach, & et al., 1998; Weakley & Schmitter-Edgecombe, 2014; Zhao et al., 2013). Earlier studies do mostly not directly compare clustering and switching within one study (but see Hirshorn & Thompson-Schill, 2006). Additionally, these studies do not directly compare clustering and switching, when strategy use is explicitly instructed. Instead, previous neuroimaging or lesion studies mainly investigated “natural” verbal fluency processes. “Natural” verbal fluency production requires both clustering and switching strategies (e.g. Bousfield & Sedgewick, 1944; Gruenewald & Lockhead, 1980; Troyer et al., 1997). It is suggested that participants first cluster words but when generation is exhausted switching occurs (Bousfield & Sedgewick, 1944; Gruenewald & Lockhead, 1980; Troyer et al., 1997). In our study the processes and resulting brain activation might be different, as they were explicitly instructed to either cluster or switch. Furthermore, Abwender, Swan, Bowerman, and Connolly (2001) proposes that switching on semantic fluency is different to phonemic fluency and is not necessarily an indication of frontal lobe strategic searching and set shifting operations on semantic fluency, whereas on phonemic fluency it is. This could further explain the lack of frontal lobe findings in the switching condition.

In the clustering condition, greater activation was also observed in the right ACC and the right superior frontal gyrus. The ACC and the DLPFC are known to reflect cognitive control processes, such as error monitoring, response conflict or inhibition (MacDonald, 2000; for a review see Botvinick, Cohen, & Carter, 2004). Activation within the SFG has also been shown in higher levels of working memory processing, like monitoring (Du Boisgueheneuc et al., 2006). For successful performance during clustering, participants need to inhibit words that come to mind from another subcategory as they are asked to produce words only from one subcategory. This may lead to stronger recruitment of these areas (ACC and SFG), as higher effort is set especially to inhibition and error monitoring compared to switching.

Irrespective of strategy and menstrual cycle phase, stronger brain activation in men compared to women was seen in several brain regions. Beside activation in the inferior parietal lobe, which is not specific to verbal fluency, areas of stronger activation in men compared to women reflect brain areas which have been shown to be important for verbal fluency performance or the execution of a language task, in general, comprising the left and right IFG/MFG, bilateral superior temporal gyrus, the left precentral gyrus the left SMA, as well as the left ACC. All these areas have been observed to be of importance during performance of verbal fluency (e.g. for a meta-analysis see Wagner et al., 2014). Hormonal analyses in the present study additionally revealed that at least for some task related areas a stronger activation was related to higher testosterone levels in men. The observation that men show stronger activation in areas important for language is comparable to results of previous studies investigating sex differences in verbal fluency and other word generation tasks (Bell et al., 2006; Gauthier et al., 2009; Gizewski et al., 2006; Halari et al., 2006; Konrad et al., 2008). As has already been discussed in previous studies, this implies that men need to recruit these areas to a stronger degree, in order to reach the same level of performance as women or to compensate for higher demands during this task. Our results further indicate that testosterone may play a key role in that respect. In line with this assumption, women show less deactivation in default mode areas like the PCC or IPL. Deactivation strength has also repeatedly been linked to task load (e.g. Newton, Morgan, Rogers, & Gore, 2011).

One new and outstanding result of the current study is, that regions with greater activation in men, for the most part, overlap to regions which are stronger activated during clustering, compared to switching. Conversely, regions of stronger activation in women compared to men mostly conform to regions of stronger activation in switching compared to clustering. This is striking insofar as previous literature on performance during verbal fluency suggests that men preferentially use the clustering strategy, whereas women switch more often between categories, compared to men (Lanting et al., 2009; Scheuringer & Pletzer, 2017; Weiss et al., 2006). Thus, our neuroimaging results strongly support previous behavioral findings of sex differences in strategy use.

Concentrating on the interaction between sex and strategy, our results suggest the right superior frontal gyrus responds with stronger activation during clustering in men, but stronger activation during switching in women. Thus for both men and women, the right superior frontal gyrus shows stronger activation for the strategy they preferred to use in previous behavioral studies. If such a sex-specific preference was also present in the current study, these results suggest an important role of the right SFG in the recognition of the preferred strategy. This area has previously been implicated in (academic) reward processing (Mizuno et al., 2008), which may explain its responsiveness to the preferred strategy in men and women.

Furthermore, not only brain activation, but also lateralization was interactively modulated by sex and strategy. Our results indicate that men indeed show stronger left-lateralization compared to women, which is in line with several previous investigations (Baxter et al., 2003; Clements et al., 2006; Jaeger et al., 1998; Kansaku & Kitazawa, 2001; McGlone, 1980; Pugh et al., 1996; Shaywitz et al., 1995; but see Frost, 1999; Weiss, Siedentopf, & et al., 2003; Sommer et al., 2004), but only during the switching condition. Additionally, left-lateralization was stronger during clustering in women, but during switching in men, suggesting a reduced lateralization of language-functions for the strategy they preferred to use in previous behavioral studies. Taken together, these results suggest that, men recruit the right hemisphere more strongly during clustering, but women during switching, which may explain why previous studies found verbal fluency performance more strongly supported by clustering in men, but by switching in women (Lanting et al., 2009; Scheuringer & Pletzer, 2017; Weiss et al., 2006). Interhemispheric cooperation between the hemispheres has been shown to be more advantageous than within-hemispheric processing in situations of high processing demands (Banich & Belger, 1990).

To follow up on this assumption, connectivity analyses were performed, which revealed that, in general, men showed primarily higher connectivity to regions within the same hemisphere of the specified ROIs, whereas women mainly show higher connectivity to regions in the contralateral hemisphere. This finding is consistent with previous findings of stronger intra-hemispheric connectivity in men, but stronger inter-hemispheric connectivity in women (Ingalhalikar et al., 2014). Hormonal analyses additionally revealed that intra-hemispheric connectivity in the right hemisphere was related to higher testosterone levels in men, and to a lesser extent also in women. This suggests that sex differences in intra-hemispheric connectivity may, at least for the right hemisphere, be hormonally mediated with testosterone playing a key role in that respect. Stronger interhemispheric processing in women on the other hand could explain the repeated finding of better performance during verbal fluency in women (Burton et al., 2005; Burton et al., 2005,; Capitani et al., 1998, 1999; Filippetti & Allegri, 2011; Halari et al., 2006; Herlitz et al., 1999; Hyde & Linn, 1988; Munro et al., 2012; Wallentin, 2009; Weiss, Kemmler, & et al., 2003; Weiss et al., 2006). Most importantly however, connectivity analyses demonstrated stronger recruitment of the right IFG and right AG by the left STG during clustering in men, but switching in women. This again supports the interpretation that the right hemisphere is more strongly recruited during clustering in men, but switching in women, i.e. the strategy they preferred to use in previous studies.

Additionally, connectivity patterns in women were similar across all ROIs. They primarily showed higher connectivity, compared to men, to bilateral frontal and parietal areas, precuneus, as well as to the cerebellum (expect for connectivity to the left SFG). The precise role of the cerebellum in language and VF is still not clear (e.g. Jansen et al., 2005). Activation within the cerebellum has repeatedly shown to be recruited during silent speech generation and synonym generation, (Jansen et al., 2005; Klein, Milner, Zatorre, Meyer, & Evans, 1995; Konrad et al., 2008; Nyberg, Forkstam, Petersson, Cabeza, & Ingvar, 2002). Conversely, in men, regions of greater connectivity, compared to women, mainly covered regions, typically involved in verbal fluency or language processing, like ipsilateral frontal, temporal and central regions, similarly to our activation results. (i.e. IFG, middle frontal, middle/superior temporal, precentral, left fusiform (only for SFG). Accordingly, for both men and women, connectivity was respectively stronger among areas they also activated during the task, albeit this was more pronounced in ipsi-lateral areas for men.

As outlined in the beginning, one limitation of the present study is that we do not have a reliable measure of performance, due to the covert task design. Nevertheless, our “rough” measure of performance indicated that irrespective of sex, participants generated more words during the clustering, compared to the switching condition. This is in line with the assumptions that switching is the more effortful condition compared to clustering, with switching being impaired under conditions of divided attention (e.g. Rende et al., 2002; Troyer et al., 1997). An additional limitation is that men and women were not tested for vocabulary as a matching criterion, though their comparable IQ and education levels do suggest similar vocabulary as well.

A strength of the present study is the large sample size and additional power gained by repeated measurements. In addition, our study design provided control for potential influences of hormone levels, as well as for possible learning effects. The present results were stable over different cycle phases and irrespective of learning effects. Session showed only limited effects on brain activation and connectivity, despite a learning effect in performance. Hormonal influences concerned in the most part testosterone levels in men, which seemed to intensify sex differences in task related areas. Interestingly, no sex differences were observed in those brain areas, for which menstrual cycle effects were previously reported (compare Supplementary Material 2), an exception being the left IFG in which menstrual cycle effects and sex differences converge. Nevertheless, even in the left IFG, sex differences supersede menstrual cycle effects with men showing stronger activation than women in all three cycle phases. These results suggest potentially different targets for organizational vs. activational effects of sex hormones (Kelly, Ostrowski, & Wilson, 1999).

To our knowledge, our study is the first directly comparing clustering vs switching during semantic fluency within one study, and the first to address sex differences in semantic fluency in an adult population. Our results support the notion that clustering and switching underlie different cognitive mechanisms and highlight differences between semantic fluency strategies and previously described phonemic fluency strategies, particularly regarding the involvement of frontal areas. In line with results from previous behavioral studies that men prefer a clustering, while women prefer a switching strategy, men show stronger activation in the clustering network, while women show stronger activation in the switching network. Furthermore, activation, lateralization and connectivity results converge in the finding that men recruit right-hemispheric areas, particularly right frontal areas more strongly during clustering, but women during switching. Finally, a more inter-hemispheric connectivity pattern in women might underlie their previously observed superior task performance. In sum, our results suggest that distinct patterns of inter-hemispheric interaction may explain previously observed sex differences in performance and strategy-use during verbal fluency.

## Supplementary Material

Supplementary data to this article can be found online at https://doi.org/10.1016/j.bandl.2020.104814

Supplementary Material

## Figures and Tables

**Table 1:** Average hormone levels in men and women during different cycle phases.

	Men	women		
		menses	Pre-ovulatory	Luteal
Estradiol [pg/ml]	1.09 ± 0.36	1.04 ± 0.43	1.37 ± 0.60*	1.16 ± 0.41
Progesterone [pg/ml]	75.80 ± 56.59	67.87 ± 41.33	83.49 ± 51.73	150.98 ± 95.08**
Testosterone [pg/ml]	120.20 ± 39.04***	62.70 ± 23.92	67.46 ± 30.36	58.49 ± 22.13

**Table 2:** Behavioral parameters for the clustering and switching instruction in men and women (averaged over the three scanning sessions).

	Men		Women	
	Clustering	Switching	Clustering	Switching
Number of words	11.44 ± 3.27	9.62 ± 2.60	12.01 ± 3.34	9.82 ± 2.59
RT [s]	2.69 ± 1.01	3.06 ± 0.70	2.56 ± 0.61	3.07 ± 0.75
Cluster size [words]	3.80 ± 1.70	0.06 ± 0.13	4.24 ± 2.55	0.10 ± 0.20
Number of switches	2.86 ± 1.14	9.41 ± 2.62	2.94 ± 1.20	9.45 ± 2.54
