# Supplementary material for: Recruiting the right hemisphere: Sex differences in inter-hemispheric communication during semantic verbal fluency
Source: Brain Lang. Author manuscript; Available in PMC 2021 Aug 30. (PMC7611590; doi:10.1016/j.bandl.2020.104814)
Supplement: Supplementary Material [file EMS131452-supplement-Supplementary_Material.zip › 1-s2.0-S0093934X20300730-mmc1.pdf]

Validation of the verbal fluency task

Participants:

45 healthy young men (mean age: 23.17 years, SD = 2.87) and 45 healthy young women (mean age: 22.13 years, SD = 2.29) participated in the validation study. All of them were right-handed German native speakers and university students, who had passed general qualification for university entrance. None of them reported any neurological, endocrinological or psychological disorders or regular use of medication. Female participants were not on hormonal contraceptives, had a regular menstrual cycle of 21-35 days according to self-reports and were tested in the luteal phase of their menstrual cycle (days 4-10 after ovulation). Ovulation was confirmed using commercial ovulation test kits (PREGNAFIX ®). Most participants also completed the screening version of the Advanced Progressive Matrices (APM; Raven et al, 1962) to assess their IQ. Men and women did not differ significantly in IQ (men: M = 106.07; SD = 13.10; women: M = 104.70; SD = 11.90; t = 0.42, p = .67) or age (t = 1.54, p = .13).

Task and Procedure:

A total of 66 verbal fluency categories was validated and each category was completed by 10 participants (5 men/5women) under neutral instructions, clustering instructions and switching instructions. To that end, three test versions were created, two containing three blocks of six categories and one containing three blocks of 10 categories (compare Table 1). 60 participants (30 men/30 women) completed the verbal fluency validation prior to another study and thus received the shorter test versions. 30 participants (15 men/15 women) only took part in the verbal fluency validation and thus received the longer test version.

Version	Block 1	Block 2	Block 3
I	insects, breakfast, beverages, professions, cities, things at the hospital	aquatic animals, sweets, containers, female names, water bodies, things at the police department	tree species, fruits, electronic devices, body parts, weather conditions, things at a construction site
II	toys, accessories, sports, metals, things in traffic, things at a farm	drugstore items, furniture, music genres, substances, things in spring, things at the fire department	clothing, art supplies, brand names, rooms, things in winter, things/people at the circus
III	mammals, dairy products, main dishes, languages, states, tools, jewelry, non-sport hobbies, gemstones, travelling	Birds, desserts, diseases, things at a hotel, mountains, writing materials, musical instruments, TV channels, colors, things in summer	flowers, vegetables, drugs, personality traits, buildings, vehicles, kitchen tools, school subjects, websites, things in autumn

Table 1. Test versions for validation of the verbal fluency task

Each version was completed by 30 participants (15 men/15 women) and the order of blocks was counterbalanced across participants, such that for each version 10 participants (5 men/5 women) completed blocks in a certain order. For the block completed first, participants always received neutral instructions. For the remaining blocks, clustering and switching instructions were alternated, such that

half of the participants received the clustering instruction first and half received the switching instruction first. For reasons of convenience, the task was completed in a computerized manner. For each category, participants received an empty word document and were asked to write down as many words as possible following the instructions within one minute. On an independent sample of 5 people, it was a priori determined for a test category ("christmas") that students are on average able to approximately type as many words in one minute as they can say in 30secs. For each category under each instruction the number of words generated, cluster size and number of switches was assessed according to the scoring rules by Troyer et al. (1997). For the switching instructions, the second word in a cluster was rated as error and did not count.

Results:

From the 66 categories, 9 were discarded due to overall difficulty (low number of words produced, e.g. gemstones), negative connotation (e.g. drugs, diseases), difficulty to identify clusters (e.g. female names) or high number of errors due to associations or strong overlap with other categories (summer, winter, spring, autumn, things at a hotel). To reach a final set of 60 categories, 3 additional categories (appetizers, garden tools, groceries) were tested only under neutral instructions for overall difficulty in an independent sample of 5 participants. Table 2 displays the 60 categories that were selected to build the final test versions.

Version1		Version2		Version3	
**Clustering**	**Switching**	**Clustering**	**Switching**	**Clustering**	**Switching**
Languages	groceries	Sports	School subjects	states	Websites
Colors	Toys	clothing	Brand names	Professions	Electr. devices
Main dishes	Beverages	Flowers	Appetizers	Ways to travel	Drugstore items
Body parts	Cities	rooms	Buildings	Vessels	Vehicles
Weather cond.	Personality traits	Circus people	Music genres	Music. instruments	Water bodies
Metals	Hobbies	Jewelry	TV channels	furniture	Textiles
Art supplies	Accessoires	Stationaries	Kitchen tools	Tools	Garden tools
Sweets	Vegetables	Fruits	Desserts	Dairy products	Breakfast
Mammals	Tree species	Insects	Aquatic animals	mountains	Birds
Things at the police station	Things in traffic	Things at a farm	Things at the hospital	Things at a construction site	Things at the fire department

Table 2. Categories included in the final test versions. Their order was pseudo-randomized for presentation.

These categories were matched across three test versions á two conditions each (clustering/switching). Average matching criteria are described in Table 3. Overall difficulty was determined by the overall number of words generated across instructions. Clustering difficulty was determined by the number of words generated in the clustering condition, as well as the average cluster size in the neutral and clustering condition. Switching difficulty was determined by the number of words generated in the switching condition, as well as the number of switches in the neutral and switching condition.

Version	Condition	Number of words			Average Cluster size			Number of switches		
		N	C	S	N	C	S	N	C	S
V1	Clustering	11.31	9.66	8.29	0.96	2.59	0.08	6.45	2.62	7.31
	Switching	11.62	10.15	7.90	0.74	2.97	0.06	6.30	2.54	7.17
V2	Clustering	10.95	9.49	7.67	0.91	2.54	0.17	6.48	2.51	6.65
	Switching	11.00	10.45	7.93	0.61	2.70	0.16	7.18	2.42	6.72
V3	Clustering	11.05	8.70	8.43	0.88	2.40	0.09	6.32	2.13	7.26
	Switching	11.24	10.30	8.15	0.79	2.53	0.11	6.40	2.60	7.18

Table 3: Matching of overall difficulty, clustering difficulty and switching difficulty across test versions
overall difficulty = number of words produced in all three categories
clustering difficulty = number of words under clustering instruction + cluster size
switching difficulty = number of words under switching instructions + number of switches
N = neutral instruction, C = clustering instruction, S = switching instruction

References

Raven, J. C., Raven, J. C. & John Hugh Court. *Advanced progressive matrices*. London: HK Lewis (1962).

Troyer, A. K., Moscovitch, M., & Winocur, G. (1997). Clustering and switching as two components of verbal fluency: Evidence from younger and older healthy adults. *Neuropsychology*, *11*(1), 138–146. https://doi.org/10.1037//0894-4105.11.1.138
