# Supplementary material for: Recruiting the right hemisphere: Sex differences in inter-hemispheric communication during semantic verbal fluency
Source: Brain Lang. Author manuscript; Available in PMC 2021 Aug 30. (PMC7611590; doi:10.1016/j.bandl.2020.104814)
Supplement: Supplementary Material [file EMS131452-supplement-Supplementary_Material.zip › 1-s2.0-S0093934X20300730-mmc2.pdf]

Supplement 2:
Sex differences in brain areas affected by menstrual cycle phase and prediction of verbal fluency performance

Background:

In the female sample, we previously tested menstrual cycle effects on the hippocampus, the caudate and the DLPFC as areas of general relevance to cognition and rich in sex hormone receptors. In the hippocampus we found stronger activation during verbal fluency in the pre-ovulatory compared to the luteal phase, while in the caudate and right DLPFC, we found stronger activation in the luteal compared to menses and pre-ovulatory phase (Pletzer et al., 2019). We also identified activation in the left hippocampus and left DLPFC as predictors of performance in the verbal fluency task. While these areas were not the main focus of our sex difference study, we also extracted eigenvalues from these areas in men to compare them to the female sample.

Statistical analyses:

Sex differences in these ROIs were assessed by the same analysis approach as sex differences in the IFG and STG, described in the main manuscript. Since menstrual cycle modulation has previously been observed, we first tested for significant sex*cycle interactions. None of the ROIs showed significant sex*cycle interactions (all $|b| < 0.39$, all $|t| < 1.72$, all $p_{FDR} > 0.48$), suggesting that sex differences did not vary along cycle phases. Accordingly sex differences were evaluated after dropping cycle from the model [formula: activation ~ session + sex*instruction + (1|PNr)]. P-values were FDR-corrected for multiple comparisons, since 6 ROIs were tested. To assess, which brain areas related to performance, a linear regression model including activation in all 6 ROIs, as described in Pletzer et al. (2019) was performed in the male sample only.

Results:

None of the ROIs showed significant sex differences or significant sex*instruction interactions (all $|b| < 0.20$, all $|t| < 1.56$, all $p_{FDR} > 0.11$). Regarding the prediction of performance in men, a significant effect of the left Hippocampus ($b = -0.27$, $SE_b = 0.14$, t = -1.96, $p = 0.05$) and left DLPFC ($b = 0.18$, $SE_b = 0.08$, $t = 2.19$, $p = 0.03$) was observed like in women. However, activation of the left hippocampus was negatively related to performance in men, i.e. men with lower hippocampal activation were able to produce more words, whereas the opposite pattern was previously observed in women (Pletzer et al., 2019). For the left DLPFC the effect went in the same direction with better performance in participants with higher DLPFC activation. Right hippocampus and right DLPFC did not significantly predict performance (both $|b| < 0.19$, both $|t| < 1.66$, both $p > 0.09$). However, unlike in women a significant negative effect of the left caudate was observed ($b = -0.36$, $SE_b = 0.10$, t = -3.33, $p = 0.001$). The higher the caudate activation, the less words were men able to produce.

References

Pletzer, B., Harris, T. A., Scheuringer, A., & Hidalgo-Lopez, E. (2019). The cycling brain: menstrual cycle related fluctuations in hippocampal and fronto-striatal activation and connectivity during cognitive tasks. Neuropsychopharmacology, 44(11), 1867-1875.
