# Supplementary material for: Recruiting the right hemisphere: Sex differences in inter-hemispheric communication during semantic verbal fluency
Source: Brain Lang. Author manuscript; Available in PMC 2021 Aug 30. (PMC7611590; doi:10.1016/j.bandl.2020.104814)
Supplement: Supplementary Material [file EMS131452-supplement-Supplementary_Material.zip › 1-s2.0-S0093934X20300730-mmc3.pdf]

Supplement 3: Learning effects

Behavioral results: Participants produced more words the more often they completed the task (b = 0.06, SE_b = 0.03, $t_{(331)}$ = 2.44, p = .02). RT, number of switches and cluster size were not affected by test session (all $|b|$ < 0.03, all SE_b > 0.01, all $|t|$ < 1.63, all p > .10).

ROI analyses: Test session did not affect activation in any ROI (all $|b|$ < 0.07, all SE_b > 0.01, all $|t|$ < 1.90, all p > .05).

Whole-brain analyses: Several areas in the task-positive network showed a negative effect of test session, indicating less activation, the more often participants completed the task (Table 1).

Brain regions	MNI-coordinates (mm)						p_{FWE} peak	p_{FWE} cluster
	Side	X	Y	Z	#voxels	*T*		
Insula	L	-39	-7	-5	301	4.92	.015	<.001
Insula	R	39	-7	2	66	3.92	.464	.025
Middle frontal gyrus	L	-21	23	4	158	4.69	.037	<.001
Middle frontal gyrus	R	33	41	4	144	4.09	.302	.001
Middle cingulate gyrus	R	-3	-22	52	76	4.08	.311	.014

Table 1. Clusters which showed significant effects of test session on brain activation.

Lateralization indices: No significant effect of session on lateralization indices was observed (b = 0.04, SE_b = 0.03, $t_{(346)}$ = 1.28, p = 0.20).

Connectivity analyses: Connectivity of the left IFG to the middle cingulate gyrus decreased with the number of test sessions ([6,-34,31], k = 96 voxels, T = 4.24, cluster p_{FWE} < 0.001, peak p_{FWE} = 0.63) – mostly white matter). No significant effects of session were observed for connectivity of the right IFG and left STG.
