# Supplementary material for: Recruiting the right hemisphere: Sex differences in inter-hemispheric communication during semantic verbal fluency
Source: Brain Lang. Author manuscript; Available in PMC 2021 Aug 30. (PMC7611590; doi:10.1016/j.bandl.2020.104814)
Supplement: Supplementary Material [file EMS131452-supplement-Supplementary_Material.zip › 1-s2.0-S0093934X20300730-mmc4.pdf]

Supplement 4: Results Tables

**Brain regions**		**MNI-coordinates**					p_{FWE}	p_{FWE}
**Clustering > Switching**	**Side**	**X**	**Y**	**Z**	**#voxels**	***T***	***peak***	***clus***
Inferior frontal gyrus	L	-48	8	19	348	5.63	.001	< .001
Inferior frontal gyrus	R	51	35	10	475	7.88	<.001	< .001
Superior frontal gyrus	R	21	32	46	112	4.93	.014	.002
Inferior temporal gyrus/inferior occipital gyrus/IPL	L	-48	-67	-8	683	11.71	<.001	<.001
Inferior temporal gyrus/inferior occipital gyrus/IPL	R	45	-58	-8	308	9.72	<.001	< .001
Superior parietal lobe	R	27	-64	37	287	7.17	<.001	< .001
Middle temporal gyrus	R	51	-7	-20	67	6.98	<.001	.023
Inferior parietal lobe	L	-60	-28	34	248	6.72	<.001	< .001
Caudate nucleus/putamen	L/R	18	11	1	448	6.80	<.001	< .001
**Switching > Clustering**								
Posterior/middle cingulate cortex	L/R	-3	-25	28	223	10.46	<.001	<.001
Precuneus/cuneus/calcarine gyrus	L/R	-9	-70	34	1904	9.44	<.001	<.001
pre/postcentral gyrus	L	-27	-7	55	286	8.91	<.001	<.001
pre/postcentral gyrus	R	27	-7	55	216	6.80	<.001	<.001
superior parietal lobe	R	33	-34	43	54	5.36	0.001	0.020
superior parietal lobe	L	-33	-52	40	103	5.16	0.001	0.002

Table 1. Clusters of significant differences in brain activation between the clustering and switching condition.

**Brain regions**		MNI-coordinates (mm)					p_{FWE}	p_{FWE}
**Women > men**	**Side**	X	Y	Z	**#voxels**	***T***	***peak***	***clus***
Pre-/postcentral gyrus/IPL	R	48	-19	16	1100	12.31	<.001	<.001
Middle temporal gyrus/Pre-/postcentral gyrus/IPL /middle cingulate cortex/Precuneus	L	-51	-55	-2	3601	12.23	<.001	<.001
Superior parietal lobe	L	-24	-61	37	105	10.91	<.001	.009
Supplementary motor area	L/R	3	14	55	87	9.35	< .001	.016
Middle frontal gyrus	R	30	53	16	60	8.30	< .001	.045
Medial prefrontal cortex	L/R	-6	35	31	77	7.89	< .001	.021
**Men > women**								
Inferior/middle frontal gyrus Precentral gyrus/supplementary motor area	L	-45	14	22	3647	19.07	<.001	<.001
Middle/superior temporal gyrus	L	-57	-34	-2	307	12.20	<.001	<.001
Middle frontal grus/ Middle/superior temporal gyrus	R	57	-22	-5	1900	14.70	<.001	<.001
Precentral gyrus	R	42	2	55	204	12.89	<.001	<.001
Inferior parietal lobe	R	27	-85	13	981	12.86	<.001	<.001
Inferior parietal lobe	L	-27	-88	13	56	10.60	<.001	.031
Posterior cingulate gyrus	L/R	-3	31	34	57	12.32	<.001	.031
Anterior cingulate gyrus	L/R	0	47	-5	137	8.34	<.001	.001
Precuneus	L	-9	-52	40	51	5.36	.001	.038

Table 2. Clusters of significant differences in brain activation between women and men

Brain regions	MNI-coordinates (mm)						p_{FWE} *peak*	p_{FWE} *clus*
**Women > men**	**Side**	**X**	**Y**	**Z**	**#voxels**	***T***		
Inferior/Middle frontal gyrus	L	-39	62	-2	156	10.98	< .001	< .001
Middle frontal gyrus	L	-33	23	37	63	6.21	< .001	.001
Superior frontal gyrus	L	-18	65	22	60	5.72	.001	.001
Inferior/Middle frontal gyrus	R	39	23	46	160	6.61	< .001	< .001
Inferior frontal gyrus	R	54	29	10	64	5.74	.001	.001
Medial prefrontal cortex	L/R	3	41	25	841	7.06	< .001	< .001
Medial prefrontal cortex	L/R	-6	8	76	67	8.60	< .001	.001
Inferior parietal lobule	L	-57	-49	37	154	8.16	< .001	< .001
Inferior parietal lobule	R	51	-64	37	291	6.90	< .001	< .001
Middle temporal gyrus	L	-57	-22	-26	58	5.86	< .001	.002
Middle temporal gyrus	R	48	-28	1	126	6.61	< .001	< .001
Precuneus	L/R	6	-52	28	614	6.96	< .001	< .001
Hippocampus	L	-27	-64	7	140	5.56	.002	< .001
Cerebellum	R	12	-88	-23	600	8.60	< .001	< .001
Cerebellum	L	-45	-64	-32	180	6.54	< .001	< .001
**Men > women**								
Inferior frontal gyrus/precentral gyrus/inferior temporal gyrus/inferior parietal lobe	L	-60	8	10	2621	14.31	< .001	< .001
Superior parietal lobe	L	-33	43	49	69	5.72	.001	.001
Inferior parietal lobe	L	-30	-67	55	100	5.06	.024	< .001
Inferior frontal gyrus	R	57	32	1	52	8.02	< .001	.003
Inferior frontal gyrus	R	45	11	22	109	5.90	< .001	< .001
Middle frontal gyrus	R	21	-1	58	166	6.12	< .001	< .001
inferior temporal gyrus	R	66	-46	1	289	6.45	< .001	< .001
Inferior parietal lobe/postcentral gyrus	R	60	-25	46	50	5.99	< .001	.004
Caudate	L	-3	17	10	132	5.72	.001	< .001

Table 3. Sex differences in connectivity patterns of the left IFG.

Brain regions	MNI-coordinates (mm)						p_{FWE} peak	p_{FWE} clus
**Women > men**	**Side**	**X**	**Y**	**Z**	**#voxels**	***T***		
Superior/middle frontal gyrus	L	-24	59	16	152	8.82	< .001	< .001
Superior/middle frontal gyrus	R	39	62	-8	179	10.46	< .001	< .001
Superior/middle frontal gyrus	L	-12	14	64	90	6.37	.001	< .001
Superior/middle frontal gyrus	R	24	23	64	55	7.63	< .001	0.003
Inferior frontal gyrus	L	-54	20	13	225	7.32	< .001	< .001
Precentral gyrus	R	36	-22	64	110	4.64	.165	< .001
Posterior/middle cingulate	L/R	0	13	37	432	7.27	< .001	< .001
Middle temporal gyrus	L	-51	-10	-29	131	6.76	< .001	< .001
Middle temporal gyrus	L	-57	-61	31	86	5.83	< .001	< .001
Inferior parietal lobe	R	54	-52	22	101	5.37	.005	< .001
Parahippocampus/Hippocampus	R	27	-37	1	168	4.57	.231	< .001
Cerebellum	L	-45	-61	-32	357	6.23	< .001	< .001
Cerebellum	R	42	-55	-32	52	5.19	.012	.004
Cerebellum	R	24	-70	-23	238	6.40	< .001	< .001
Cerebellum	L/R	3	-64	-14	159	4.44	.371	< .001
**Men > women**								
Inferior frontal gyrus/precentral gyrus	R	63	11	10	485	11.98	< .001	< .001
Middle frontal gyrus	R	36	47	31	633	6.84	< .001	< .001
Superior frontal gyrus/precentral gyrus	R	18	-22	82	331	6.89	<.001	<.001
Superior temporal gyrus	R	63	-34	16	105	8.53	< .001	< .001
Middle temporal gyrus	R	51	-52	1	71	6.22	< .001	< .001
Middle temporal gyrus	L	-39	-70	13	87	5.30	.007	< .001
Putamen	L	-27	20	4	66	5.11	.019	.001
Insula	L	-42	2	-2	63	4.68	.139	.001

Table 4. Sex differences in connectivity patterns of the right IFG.

Brain regions		MNI-coordinates (mm)					p_{FWE}	p_{FWE}
**Women > men**	**Side**	**X**	**Y**	**Z**	**#voxels**	***T***	***peak***	***clus***
Superior/middle frontal gyrus	R	27	56	13	426	6.89	< .001	< .001
Superior/middle frontal gyrus	L	-27	53	19	112	4.60	.195	< .001
Insula	L	-36	2	-17	51	6.59	< .001	.005
Lingual gyrus	R	9	-31	-11	67	5.79	.001	.001
Inferior parietal lobule	L	-66	-31	22	121	10.48	< .001	.001
Inferior parietal lobule	R	60	-46	22	628	7.82	< .001	< .001
Posterior cingulate gyrus	L/R	6	-34	49	669	7.01	< .001	< .001
**Men > women**								
Inferior frontal gyrus	L	-51	26	-5	532	9.13	< .001	< .001
Middle frontal gyrus	L	-36	20	58	69	6.57	< .001	.001
Middle frontal gyrus	L	-51	32	31	176	5.38	.005	< .001
Middle frontal gyrus	R	33	65	1	50	5.78	.001	.005
Precentral gyrus	R	54	-1	46	72	6.99	< .001	.001
Middle temporal gyrus	L	-66	-37	-2	875	8.85	< .001	< .001
Middle temporal gyrus	L	-63	-4	-20	72	5.97	< .001	.001
Inferior parietal lobe	L	-36	-73	46	64	5.76	.001	.001
Cuneus	R	24	-79	13	97	5.55	.002	< .001
Supplementary motor area	L/R	-6	11	52	64	5.32	.006	.001
Cerebellum	R	33	-70	-38	92	5.23	.010	< .001

Table 5. Sex differences in connectivity patterns of the left STG.
